# Supplementary material for: A Likelihood Approach for Real-Time Calibration of Stochastic Compartmental Epidemic Models
Source: PLoS Comput Biol. 2017 Jan 17;13(1):e1005257. doi: 10.1371/journal.pcbi.1005257 (PMC5240920; doi:10.1371/journal.pcbi.1005257)
Supplement: S1 File — (TAR.GZ) [file pcbi.1005257.s014.tar.gz › HSPH_Online-SI-Revision/IPoi09/n1-severe/n1-severe_BENCH_summary.pdf]

| peak -8 | R0 | $1/\theta_2$ | inf | inflog | newly | newlylog | Reff | AttackRate |
|---|---|---|---|---|---|---|---|---|
| mean | 28.5 | 51.3 | 161.4 | 0.5 | 242. | 0.6 | 30.6 | 40. |
| median | 26.1 | 39.2 | 111.4 | 0.5 | 141.1 | 0.6 | 29.1 | 40. |
| Quantile .1 | 12. | 22.5 | 59.6 | 0.4 | 73.4 | 0.4 | 15.6 | 20.8 |
| Quantile .9 | 43.1 | 74.7 | 297.8 | 0.7 | 411.2 | 0.9 | 44.1 | 58.4 |
| 36 | 35 | 36 | | | | | | |
| length | 50 | | | | | | | |

| peak -4 | R0 | $1/\theta_2$ | inf | inflog | newly | newlylog | Reff | AttackRate |
|---|---|---|---|---|---|---|---|---|
| mean | 29.2 | 48.3 | 114.4 | 0.4 | 86. | 0.3 | 31.3 | 30. |
| median | 26.3 | 39.8 | 94. | 0.4 | 71. | 0.3 | 30.1 | 27.7 |
| Quantile .1 | 6.5 | 15. | 48.3 | 0.2 | 36.3 | 0.2 | 20.9 | 13.3 |
| Quantile .9 | 53. | 93. | 208.8 | 0.5 | 141.5 | 0.4 | 41.2 | 46.7 |
| 16 | 23 | 12 | | | | | | |
| length | 50 | | | | | | | |

| peak | R0 | $1/\theta_2$ | inf | inflog | newly | newlylog | Reff | AttackRate |
|---|---|---|---|---|---|---|---|---|
| mean | 50.1 | 71.3 | 136.8 | 0.4 | 58.3 | 0.2 | 36.9 | 40.4 |
| median | 49.9 | 52.7 | 123.3 | 0.3 | 50.5 | 0.2 | 37.1 | 39.9 |
| Quantile .1 | 21.9 | 12.6 | 37.7 | 0.2 | 25.4 | 0.1 | 28.2 | 25.7 |
| Quantile .9 | 76.7 | 170. | 255.8 | 0.5 | 104.1 | 0.3 | 44. | 58.8 |
| 0 | 5 | 0 | | | | | | |
| length | 50 | | | | | | | |

| peak +4 | R0 | $1/\theta_2$ | inf | inflog | newly | newlylog | Reff | AttackRate |
|---|---|---|---|---|---|---|---|---|
| mean | 53.5 | 73.7 | 204.9 | 0.4 | 79.3 | 0.2 | 43.8 | 41.8 |
| median | 52.7 | 59.9 | 123.1 | 0.3 | 56.8 | 0.2 | 44.2 | 40.9 |
| Quantile .1 | 24.9 | 7.6 | 42.7 | 0.2 | 37.4 | 0.2 | 34.6 | 25.9 |
| Quantile .9 | 81.6 | 171.9 | 428.4 | 0.7 | 148.8 | 0.4 | 52.2 | 55.9 |
| 0 | 5 | 0 | | | | | | |
| length | 50 | | | | | | | |

| peak +8 | R0 | $1/\theta_2$ | inf | inflog | newly | newlylog | Reff | AttackRate |
|---|---|---|---|---|---|---|---|---|
| mean | 54.9 | 73.6 | 268 014. | 0.5 | 1050.2 | 0.4 | 50.1 | 42.7 |
| median | 53.3 | 62.4 | 130.6 | 0.3 | 71.4 | 0.3 | 49.1 | 42.5 |
| Quantile .1 | 23.9 | 7.7 | 46.8 | 0.2 | 45.2 | 0.2 | 37.7 | 25.5 |
| Quantile .9 | 78.5 | 151.5 | 711.1 | 0.8 | 574.9 | 0.7 | 62.2 | 58. |
| 0 | 1 | 0 | | | | | | |
| length | 50 | | | | | | | |
